# Supplementary material for: Resolving the full spectrum of human genome variation using Linked-Reads
Source: Genome Res. 2019 Apr;29(4):635–45. doi: 10.1101/gr.234443.118 (PMC6442396; doi:10.1101/gr.234443.118)
Supplement: Supplemental Material [file supp_gr.234443.118_Supplemental_longranger-2.2.zip › longranger-2.2.tar.bz2!/lr2.2-public/lib/stan/cmdstan/stan/doc/api/html/vari_8hpp_source.html]

Stan: src/stan/agrad/rev/vari.hpp Source File


|  |  |
| --- | --- |
|  | Stan  2.5.0  probability, sampling & optimization |


- Main Page
- Related Pages
- Namespaces
- Classes
- Files

- File List
- File Members

All Classes Namespaces Files Functions Variables Typedefs Enumerations Enumerator Friends Macros Pages

- src
- stan
- agrad
- rev

vari.hpp

Go to the documentation of this file.

1 #ifndef STAN\_\_AGRAD\_\_REV\_\_VARI\_HPP

2 #define STAN\_\_AGRAD\_\_REV\_\_VARI\_HPP

3

4 #include <ostream>

5

6 #include <stan/agrad/rev/var\_stack.hpp>

7 #include <stan/agrad/rev/chainable.hpp>

8

9 namespace stan {

10  namespace agrad {

11

12  // forward declaration of var

13  class var;

14

28  class vari : public chainable {

29  private:

30  friend class var;

31

32  public:

33

37  const double val\_;

38

43  double adj\_;

44

57  vari(const double x):

58  val\_(x),

59  adj\_(0.0) {

60  var\_stack\_.push\_back(this);

61  }

62

63  vari(const double x,bool stacked):

64  val\_(x),

65  adj\_(0.0) {

66  if (stacked)

67  var\_stack\_.push\_back(this);

68  else

69  var\_nochain\_stack\_.push\_back(this);

70  }

71

79  virtual ~vari() {

80  // throw std::logic\_error("vari destruction handled automatically");

81  }

82

88  virtual void init\_dependent() {

89  adj\_ = 1.0; // droot/droot = 1

90  }

91

95  virtual void set\_zero\_adjoint() {

96  adj\_ = 0.0;

97  }

98

108  friend std::ostream& operator<<(std::ostream& os, const vari\* v) {

109  return os << v << " " << v->val\_ << " : " << v->adj\_;

110  }

111  };

112

113  }

114 }

115 #endif

var\_stack.hpp

stan::agrad::vari::init\_dependent

virtual void init\_dependent()

Initialize the adjoint for this (dependent) variable to 1.

**Definition:** vari.hpp:88

stan::agrad::chainable

Abstract base class for variable implementations that handles memory management and applying the chai...

**Definition:** chainable.hpp:14

chainable.hpp

stan::agrad::vari::val\_

const double val\_

The value of this variable.

**Definition:** vari.hpp:37

stan::agrad::vari::vari

vari(const double x, bool stacked)

**Definition:** vari.hpp:63

stan::agrad::vari::vari

vari(const double x)

Construct a variable implementation from a value.

**Definition:** vari.hpp:57

stan::agrad::vari

The variable implementation base class.

**Definition:** vari.hpp:28

stan::agrad::var\_stack\_

std::vector< chainable \* > var\_stack\_

**Definition:** var\_stack.cpp:13

stan::agrad::vari::~vari

virtual ~vari()

Throw an illegal argument exception.

**Definition:** vari.hpp:79

stan::agrad::var

Independent (input) and dependent (output) variables for gradients.

**Definition:** var.hpp:27

stan::agrad::vari::set\_zero\_adjoint

virtual void set\_zero\_adjoint()

Set the adjoint value of this variable to 0.

**Definition:** vari.hpp:95

stan::agrad::vari::adj\_

double adj\_

The adjoint of this variable, which is the partial derivative of this variable with respect to the ro...

**Definition:** vari.hpp:43

stan::agrad::var\_nochain\_stack\_

std::vector< chainable \* > var\_nochain\_stack\_

**Definition:** var\_stack.cpp:14

stan::agrad::vari::operator<<

friend std::ostream & operator<<(std::ostream &os, const vari \*v)

Insertion operator for vari.

**Definition:** vari.hpp:108


---

[ Stan Home Page ]

*© 2011–2014,
Stan Development Team.*
